# Histone H3 lysine 4 methylation is associated with the transcriptional reprogramming efficiency of somatic nuclei by oocytes

**DOI:** 10.1186/1756-8935-3-4

**Published:** 2010-02-04

**Authors:** Kazutaka Murata, Tony Kouzarides, Andrew J Bannister, John B Gurdon

**Affiliations:** 1 The Wellcome Trust/Cancer Research UK Gurdon Institute, University of Cambridge, Tennis Court Road, Cambridge CB2 1QN, UK; 2 Department of Zoology, University of Cambridge, Cambridge, UK; 3 Department of Pathology, University of Cambridge, Cambridge, UK

## Abstract

**Background:**

When the nuclei of mammalian somatic cells are transplanted to amphibian oocytes in the first meiotic prophase, they are rapidly induced to begin transcribing several pluripotency genes, including Sox2 and Oct4. The more differentiated the donor cells of the nuclei, the longer it takes for the pluripotency genes to be activated after the nuclear transfer to oocytes. We have used this effect in order to investigate the role of histone modifications in this example of nuclear reprogramming.

**Results:**

Reverse transcription polymerase chain reaction analysis shows that the transcriptional reprogramming of pluripotency genes, such as Sox2 and Oct4, takes place in transplanted nuclei from C3H10T1/2 cells and from newly differentiated mouse embryonic stem cells. We find that the reprogramming of 10T1/2 nuclei is accompanied by an increased phosphorylation, an increased methylation and a rapidly reduced acetylation of several amino acids in H3 and other histones. These results are obtained by the immunofluorescent staining of transplanted nuclei and by Western blot analysis. We have also used chromatin immunoprecipitation analysis to define histone modifications associated with the regulatory or coding regions of pluripotency genes in transplanted nuclei. Histone phosphorylation is increased and histone acetylation is decreased in several regulatory and gene coding regions. An increase of histone H3 lysine 4 dimethylation (H3K4 me2) is seen in the regulatory regions and gene coding region of pluripotency genes in reprogrammed nuclei. Furthermore, histone H3 lysine 4 trimethylation (H3K4 me3) is observed more strongly in the regulatory regions of pluripotency genes in transplanted nuclei that are rapidly reprogrammed than in nuclei that are reprogrammed slowly and are not seen in β-globin, a gene that is not reprogrammed. When 10T1/2 nuclei are incubated in *Xenopus *oocyte extracts, histone H3 serine 10 (H3S10) is strongly phosphorylated within a few hours. Immunodepletion of Aurora B prevents this phosphorylation.

**Conclusion:**

We conclude that H3K4 me2 and me3 are likely to be important for the efficient reprogramming of pluripotency genes in somatic nuclei by amphibian oocytes and that Aurora B kinase is required for H3S10 phosphorylation which is induced in transplanted somatic cell nuclei.

## Background

The transcriptional status of differentiated cells can be reversed by somatic cell nuclear transfer to eggs. It is known that mouse somatic cell nuclei transplanted to *Xenopus laevis *oocyte germinal vesicles (GVs) undergo transcriptional reprogramming in order to express pluripotency genes, including Oct4 that are not expressed in normal adult somatic cells [1]. The reprogramming of mouse somatic cell nuclei by *X*. oocyte GVs provides a unique opportunity to analyse the fundamental mechanisms that accompany transcriptional reprogramming without DNA replication and cell division.

Although it is known that epigenetic changes occur extensively during nuclear reprogramming [2,3], the mechanisms of reprogramming remain unknown. The transplantation of mammalian somatic cell nuclei to amphibian oocytes in the first meiotic prophase provides an opportunity to analyse nuclear reprogramming in ways not accessible in other kinds of nuclear transfer experiments. First, the absence of DNA replication and cell division in oocytes eliminates the possibility that changes seen in nuclei transplanted to eggs could be related to replication and not transcription. Second, the large number of nuclei transplanted to an oocyte makes it possible to investigate chromatin changes by Western blot and chromatin immunoprecipitation (ChIP) analyses. We have taken advantage of these opportunities to find changes in histone H3 modifications that accompany the transcriptional activation of pluripotency genes.

Previous work has described histone modifications associated with nuclei transplanted to mammalian eggs [4,5], with embryonic (ES) hybrid cells [3], or with induced pluripotent stem cells [6]. However, histone modifications at the chromatin level in transplanted nuclei as they are transcriptionally reprogrammed have not before been investigated.

It is thought that the reprogramming potential of donor nuclei may be lost as cells differentiate progressively in frogs or mice. This is because there is an inverse relationship between embryo survival rates and the increasing differentiation of donor cells. Donors taken from earlier developmental stages can elicit more normal development than ones from later stages [7,8]. However, despite efforts to understand the underlying mechanisms of this phenomenon, it still remains unclear which epigenetic changes are related to transcriptional reprogramming efficiency. DNA methylation may influence the efficiency of cloning in mice [9].

In this study, we have investigated a range of histone modifications in mouse nuclei transplanted to X. oocyte GVs in order to ask whether histone modifications are changed at the nuclear or chromatin levels. We find that histone H3 phosphorylations and H3/H4 methylations increase but histone H3/H4 acetylations decrease, as nuclei undergo reprogramming. These changes are found by ChIP assay to apply to the promoter and gene coding regions of pluripotency genes. Our results extend what is known of nuclear reprogramming by showing a relationship between H3K4 methylation and the transcriptional activation of the pluripotency genes Sox2, Oct4 and Sall4.

## Results

### Changes that accompany the reprogramming of transplanted nuclei can be assessed by immunofluorescent staining, Western blot analysis andChIP

**Figure 1:**
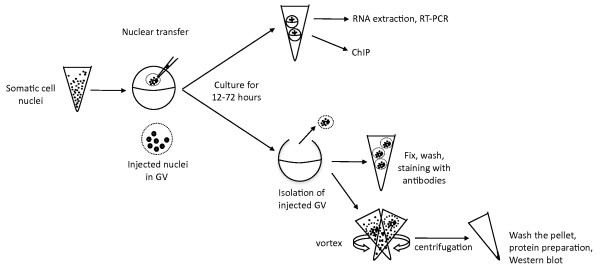
**Procedures for immunofluorescent staining and Western blotting on mouse somatic nuclei transplanted to *Xenopus laevis *oocyte germinal vesicles (GVs)**. Mouse somatic cells were permeabilized and injected to GVs. Injected oocytes were cultured for the desired time. Whole oocytes were collected for isolating RNA or for chromatin immunoprecipitation assay. For immunofluorescent staining or Western blotting, injected oocytes were manually dissected to isolate GVs. The isolated GVs were either fixed to be stained or dispersed by vortexing to collect only injected somatic nuclei.

We have used several procedures to examine the transcriptional reprogramming of pluripotency genes and histone modifications in transplanted nuclei. One is to isolate RNA from amphibian oocytes injected with mouse cell nuclei for reverse transcription polymerase chain reaction (RT-PCR) analysis of transcriptional activation (Figure 1). The second is to manually isolate the GVs of injected oocytes, fix their contents, including the injected somatic nuclei, and stain by antibodies (Figure 1). This procedure shows the presence of modified histones in individual injected nuclei. It therefore gives a measure of the proportion of injected nuclei that contain modified histones, as well as an indication of their abundance in each injected nucleus. Our third procedure (Figure 1) is to separate transplanted nuclei from injected GVs and to carry out a Western blot analysis of modified histones using the same range of antibodies as before. This procedure identifies more accurately the modified histones of injected nuclei and gives a more precise estimate of their average abundance or deficiency. Our fourth procedure is to carry out ChIP in order to investigate histone modifications of the chromatin in specific regions of pluripotency genes (Figure 1). It is technically feasible to analyse histone modifications in transplanted nuclei by these complementary procedures because of the large number of nuclei (typically 200) injected per oocyte.

### The transcriptional activation of pluripotency genes is delayed as donor cells become more differentiated

**Figure 2:**
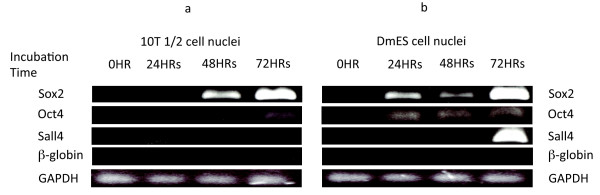
**Reverse transcription polymerase chain reaction analysis of embryonic stem (ES) cell marker genes in transplanted 10T1/2 cell nuclei and mouse ES cell nuclei**. 10T1/2 cell nuclei (a) or differentiated mouse ES cell nuclei (b) were injected into *Xenopus *oocyte germinal vesicles and incubated at 14°C for the times indicated. Expression of Sox2, Oct4 and Sall4 were analysed as pluripotency genes. Expression of β-globin was tested as a non-reprogrammed gene. GAPDH expression was examined as a reference gene.

For the experiments reported here, we have chosen to use nuclei from 10T1/2 cells and to compare these results with those of nuclei from DmES cells. 10T1/2 cells are partially differentiated to the extent that they are committed to form only certain types of mesodermal tissue [10,11]. When 10T1/2 cell nuclei are transplanted to *X*. oocytes, Sox2 transcription is not seen strongly until 48 h and Oct4 transcription only weakly at 72 h (Figure 2a). In contrast, the nuclei of DmES cells express Sox2 (and Oct4 more weakly) from 24 h (Figure 2b). They express Sall4 strongly at 72 h (Figure 2b). Neither cell type is induced to express β-globin at any time (Hbb-1 and Hbb-2) [12]. This more rapid activation of pluripotency genes in nuclei from newly committed DmES than from more committed 10T1/2 cells, as we now see in oocytes, is reminiscent of previous experience with single nuclear transfers to eggs. In egg nuclear transfers the nuclei of embryonic (blastula) cells yield much more normal development than the nuclei of differentiated (tadpole) cells [7]. Therefore, the present results indicate that the efficiency of pluripotency gene reactivation by oocytes depends on the state of donor nuclei. This difference in the rate of reprogramming between 10T1/2 and DmES cell nuclei helps us to judge the significance of the epigenetic changes that we now describe.

### Histone phosphorylation and methylation are increased, and acetylation reduced, in nuclei transplanted to oocytes

**Figure 3:**
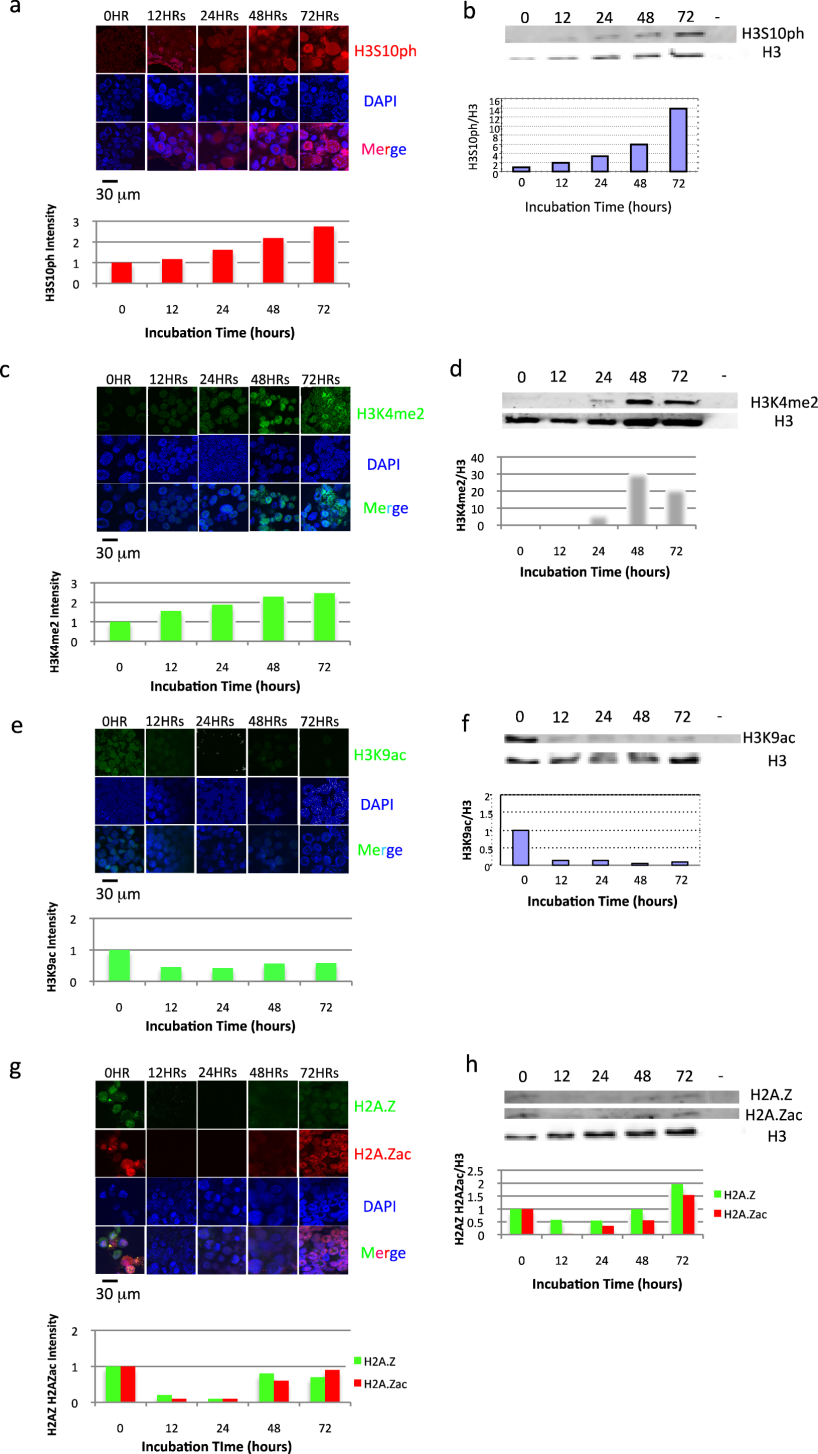
**Global changes in histone modifications in somatic cell nuclei injected to germinal vesicles (GVs)**. GVs containing transplanted 10T1/2 nuclei were isolated from oocytes. The isolated GVs with nuclei were fixed by 4%PFA and stained by antibodies against H3S10ph as shown in red (a), H3K4 me2 shown in green (c), H3K9ac shown in green (e), H2A.Z shown in green (g) and H2A.Zac shown in red (g). All nuclei were counterstained by DAPI shown in blue. The intensity of staining, which was measured by ImageJ, is shown as columns below the pictures. Re-isolated nuclei were also subject to Western blotting, shown in b, d, f and h. In each case, the far right panel which has no signal is from GVs without transplanted 10T1/2 cell nuclei. In each case the values for modified histones were normalized by reference to values for H3 histones.

The phosphorylation of histone H3 serine 10 (H3S10ph) takes place in 10T1/2 nuclei gradually and progressively over 3 days (Figure 3a and 3b). The results of immunofluorescent staining of whole injected nuclei and of Western blot analyses are in good agreement. A progressive increase in other histone H3 phosphorylations such as H3T3ph, H3T6ph, and H3T11ph are also seen (Additional File 1). Many different histone methylations also show a progressive increase in transplanted nuclei. A good example is that of H3K4 me2 (Figure 3c and 3d). Other examples are seen in Additional File 1.

At the same time as histone H3 phosphorylations and methylations increase, we see a rapid decrease in histone acetylation such as H3 lysine 9 acetylation (Figure 3e and 3f). Another histone acetylation, namely H3 lysine 14, also decreases (Additional File 1). This decrease also applies to H2A.Z acetylation (H2A.Zac), although there is a subsequent increase in H2A.Zac and H2A.Z (Figure 3g and 3h). All results of immunofluorescent staining of transplanted 10T1/2 are summarized in Additional File 1.

We conclude that *X*. oocytes induce changes in histone modifications in injected somatic nuclei. In most cases, the histone changes in transplanted nuclei take place on a slow, progressive time scale, with the exception of H3K9 deacetylation and H2A.Z extinction which take place very rapidly.

### Histone modifications are associated with the regulatory regions of pluripotency genes

**Figure 4:**
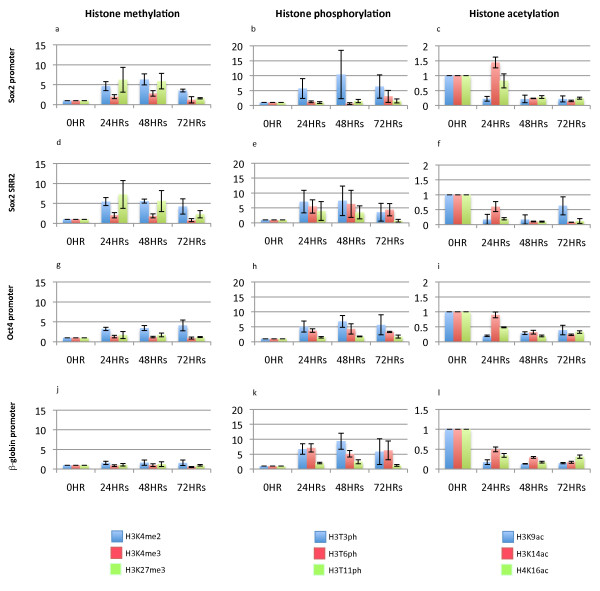
**Chromatin immunoprecipitation analyses of histone modifications in 10T1/2 cell nuclei transplanted to germinal vesicles**. Histone methylations, phosphorylations and acetylations in the promoter (a, b and c) or regulatory regions (d, e and f) of Sox2, Oct4 (g, h and i) and β-globin (j, k and l) in transplanted 10T1/2 nuclei were analyzed. H3K4 me2, H3T3ph and H3K9ac are shown in blue; H3K4 me3, H3T6ph and H3K14ac are shown in red; H3K27 me3, H3T11ph, and H4K16ac are shown in light green. The *Y *axis is the fold enrichment of modified histone over non-modified histone H3. The *X *axis is the incubation time after nuclear transfer.

In order to investigate the significance of histone modification changes in specific regions of Sox2, Oct4 and β-globin (Figure 4) in transplanted nuclei, we have conducted ChIP analysis. We have compared the enrichment of histone modifications on the pluripotency genes Sox2 and Oct4 to those on β-globin, a gene that shows no transcriptional activation in injected oocytes [12]. Positions of primers for this analysis are shown in Additional File 2. β-globin shows a rapid increase in histone phosphorylation and decrease in histone acetylation in its promoter region (Figure 4k and 4l) as is also observed in the promoter and regulatory regions of Sox2 and Oct4 (Figure 4b, c, e, f, h and 4i). This suggests that the phosphorylations and deacetylations that are seen are a general response of injected nuclei to an oocyte and are not determinative for reprogramming. They may be necessary, but are not sufficient.

On the other hand, changes in histone methylation seem more likely to be associated with reprogramming. A rapid and strong increase (approximately fivefold) in H3K4 me2 and slight increase (about double) in H3K4 me3 are seen in the promoter and regulatory regions of Sox2 (Figure 4a and 4d). For Oct4, H3K4 me2 increases in the promoter (Figure 4g). A rapid and strong increase of H3K27 me3 is also found in the Sox2 promoter and regulatory regions (Figure 4a and 4d). No such methylation increase is seen in the promoter region of β-globin (Figure 4j).

In respect of gene coding regions, the following changes are seen. β-globin shows a rapid increase in histone phosphorylation and decrease in histone acetylation (Additional File 3h and 3i), as is also observed in Sox2 and Oct4 (Additional File 3b, c, e and 3f). However, a progressive and robust increase (approximately eightfold) in H3K4 me2 is observed in Sox2 gene coding region (Additional File 3a), possibly for active transcription [13,14]. In the Oct4 gene coding region, H3K4 me2 increases as well as H3K27 me3 (Additional File 3d). It is important to note that the transcriptional activation of Sox2 is much faster than that of Oct4 (Figure 2a), in agreement with the rapidity of these histone H3 methylation changes. We conclude that changes of H3K4 me2 in the coding regions of pluripotency genes agree well with their active transcription in transplanted 10T1/2 cell nuclei (Figure 2a).

### A comparison between 10T1/2 and DmES nuclei suggests the likely importance of H3K4 trimethylation for efficient transcriptional reprogramming

To further test the significance of the histone H3 methylations so far described for 10T1/2 nuclei, we have examined similar changes in DmES cell nuclei. As mentioned above, these DmES nuclei activate pluripotency genes much more rapidly after transfer to oocytes than do 10T1/2 nuclei (Figure 2). We have paid special attention to histone H3 methylations (H3K4 me2, H3K4 me3 and H3K27 me3) because these seem from our 10T1/2 nuclei results to be likely to be connected with reprogramming.

**Figure 5:**
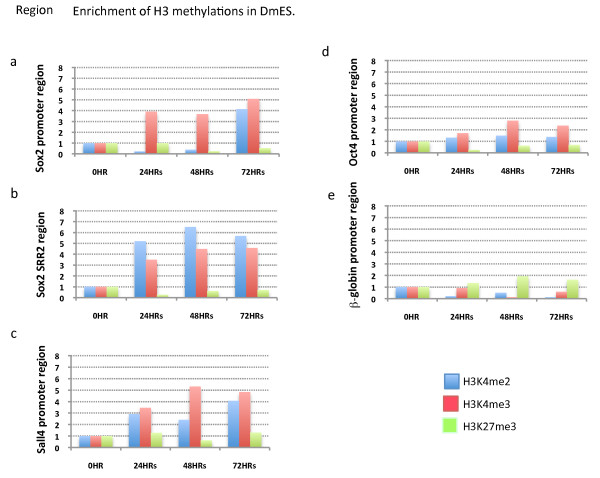
**Chromatin immunoprecipitation analyses of histone modifications in differentiated mouse embryonic stem (DmES) cell nuclei transplanted to *Xenopus *germinal vesicles**. Histone methylations in the promoter or regulatory regions of Sox2 (a and b), Sall4 (c), Oct4 (d) and β-globin (e) in transplanted DmES nuclei were analysed. H3K4 me2 is shown in blue, H3K4 me3 in red and H3K27 me3 in light green. The *Y *axis is the fold enrichment of modified histone over non-modified histone H3. The *X *axis is incubation time after nuclear transfer.

The first clear result with DmES nuclei is that we see no significant changes to histone H3K4 methylation but a weak increase of H3K27 me3 in the β-globin promoter (Figure 5e), a negative control in agreement with what is seen with globin in 10T1/2 nuclei (Figure 4j). An increase in H3K4 me3 takes place in the promoter regions of Sox2 and Sall4 as well as in the Sox2 regulatory region within 24 hours (Figure 5a-c). An increase in H3K4 me2 is seen in the Sox2 regulatory region and in the Sall4 promoter in DmES nuclei (Figure 5a-c). The most significant difference between DmES and 10T1/2 nuclei is an increase of H3K4 me3 in the Sox2, Sall4 and Oct4 promoter and regulatory regions. The increment of H3K4 me3 is substantial in Sox2 and Sall4 promoter region only in DmES nuclei (Figure 5a-c). The increase of H3K4 me3 in the Oct4 promoter region in DmES nuclei is weaker than the same change in the Sox2 and Sall4 promoter and regulatory regions. However this change is still twice as strong in DmES nuclei as in 10T1/2 cell nuclei (Figures 5d and 4g). Another considerable difference between these nuclei is the level of H3K27 me3. In 10T1/2 nuclei, H3K27 me3 strongly increases in the Sox2 promoter (Figure 4a), in the Sox2 regulatory region (Figure 4d) and, weakly, in Oct4 promoter (Figure 4g). However, this increase is not observed in DmES nuclei (Figure 5a, b and 5d). These results may explain why 10T1/2 nuclei show a retarded transcriptional reprogramming of pluripotent genes because their promoter and regulatory regions form bivalent domains in 10T1/2 nuclei, but the strong increase of H3K4 me3 without an H3K27 me3 increase may result in more efficient reprogramming in DmES nuclei.

### Aurora B kinase has a phosphorylating activity in oocytes

**Figure 6:**
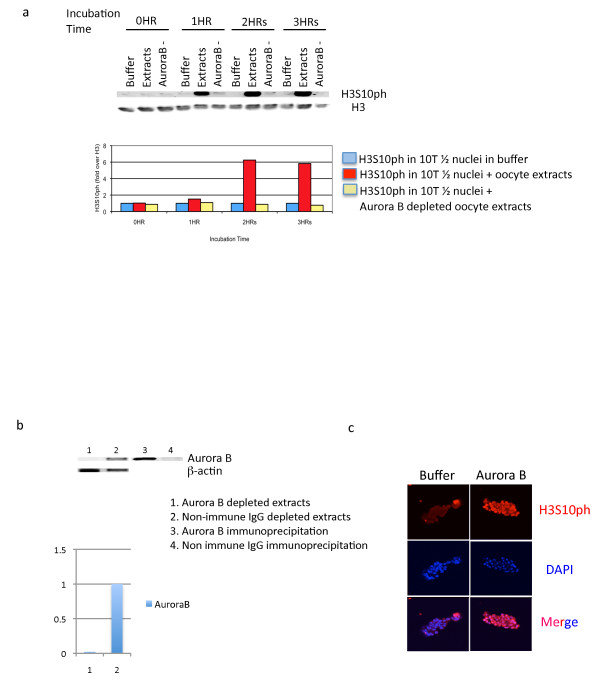
**Aurora B kinase is required and sufficient to phosphorylate H3S10ph in 10T1/2 cell nuclei transplanted to germinal vesicles**. (a) A Western blot of H3S10ph in nuclei incubated in buffer, oocyte extracts or in Aurora B immunodepleted oocyte extracts is shown. The quantitative analysis of these bands is shown below the Western blot; H3S10ph normalized to H3 in nuclei incubated in buffer is shown in blue, in oocyte extracts is shown in red and in nuclei in depleted extracts in yellow.(b) Aurora B immunodepletion from oocyte extracts. The level of Aurora B protein in Aurora B depleted oocyte extracts (lane 1), in non-depleted extracts (lane 2), in depleted Aurora B (lane3), or non-depleted Aurora B (lane 4) is shown. β-actin was also examined as a reference protein. The bar-chart shows the ratio of Aurora B:β-actin. (c) Aurora B protein can phosphorylate H3S10 in 10T1/2 nuclei. 10T1/2 nuclei were incubated with Aurora B protein for 1 h. H3S10ph is shown in red. 10T1/2 nuclei are stained with DAPI and shown in blue.

The availability of anti-Aurora B [15] antibodies gives us an opportunity to investigate the oocyte activity for H3S10ph in transplanted nuclei. We have been able to reproduce H3S10ph by adding oocyte extracts to our standard preparation of 10T1/2 nuclei. A strong phosphorylation of H3S10 is seen within a few hours of adding an oocyte extract, but not if the extract has been immunodepleted by antibody (Figure 6a). As H3S10 phosphorylation is successful in oocyte extracts, we have used these extracts for immunodepletion. The immunodepletion used is successful in removing the Aurora B kinase activity (Figure 6b). Furthermore, H3S10ph increases in 10T1/2 cell nuclei when these cell nuclei are incubated with Aurora B protein (Figure 6c). We conclude that oocytes have an active Aurora B kinase that is responsible, among other functions, for phosphorylating appropriate parts of H3 histone. The inhibitor ZM447439 [16] had a toxic effect on oocytes, thereby precluding the injection of nuclei into oocytes lacking Aurora B kinase.

## Discussion

Nuclear reprogramming is of interest because it reverses what is normally a highly stable state of cell differentiation. Furthermore, it opens up the eventual possibility of deriving therapeutically useful cells of a desired kind from easily accessible cells (such as skin) of the same individual, leading to immunologically tolerant cell replacement. At present, there are three ways in which differentiated cells can be experimentally reprogrammed. Cell fusion can efficiently re-set gene expression in adult lymphocytes [17] but it is difficult to remove the host cell nucleus from the fused (usually tetraploid) cell. Induced pluripotency [18] helps to create embryonic stem cells and, hence, a range of differentiated cell types, from differentiated adult cells but the efficiency is low and the mechanism of reprogramming may well be stochastic [19]. Nuclear transfer to eggs (M2 metaphase) and oocytes (M1 prophase) makes use of a natural reprogramming activity of these cells but its potential therapeutic value is greatly restricted because of the difficulty of obtaining a supply of human eggs. For this reason, it is very desirable to identify the mechanisms and molecules used in nuclear reprogramming by eggs in order to eventually take advantage of their natural reprogramming components. The results reported here take a step in this direction by identifying epigenetic modifications associated with nuclear reprogramming by oocytes.

In this work we have studied reprogramming by injecting multiple somatic nuclei into mature oocytes in first meiotic prophase. Compared to eggs in the second meiotic metaphase, oocytes have several advantages. They are wholly inactive in DNA synthesis, whereas eggs are very active in DNA synthesis but inactive in transcription. Nuclei injected into oocytes start to transcribe previously quiescent pluripotency genes within 24 h at 14°C (equivalent to 6 h at a mammalian temperature). The reprogrammed nuclei do not generate new cells, but are valuable for elucidating the processes that accompany this transcriptional activation of pluripotency genes in the complete absence of cell division or DNA replication.

Previous accounts of histone modifications in nuclei transplanted to mouse eggs (second meiotic metaphase) have used antibody staining of fixed material [4]. In this case, a rapid deacetylation of H3K9, H3K14 and H4K16, with a subsequent reacetylation, was seen but no change in other histones (H4K8, H4K12). The same analysis saw a gradual demethylation of H3K9 me2 and me3. It was not possible, in this case, to know whether the histone changes seen were associated with chromatin or with identified genes and these changes could be related to the cell cycle or to DNA replication. Using antibody staining of transplanted nuclei in fixed GV stage mouse oocytes (first meiotic prophase), Bui *et al*. [5] found that H3K9 is fully demethylated, and H3K9 and H3K14 are partially deacetylated. However, the activities described by Bui *et al*. [5] are located primarily in the cytoplasm of oocytes; the *Xenopus *activities described here are largely restricted to the GV, known from our previous work to be very effective in transcriptional reactivation [1,12].

**Figure 7:**
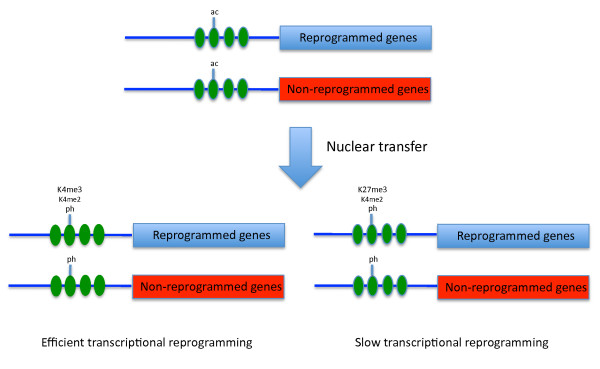
**Schematic illustration of histone modification changes during transcriptional reprogramming in mouse somatic cell nuclei transplanted to *Xenopus *germinal vesicles**. A green oval represents nucleosomes. Horizontal blue bars represent DNA in promoter regions. Blue bars represent genes that are reprogrammed, such as Sox2 or Oct4. Red bars represent genes that are not reprogrammed, such as β-globin. Short vertical bars representing histone tails and modifications that take place in each condition are shown above the tails. ac indicates various histone acetylations; ph indicates various histone phosphorylations; K4 me2 indicates H3K4 me2; K4 me3 indicates H3K4 me3; and K27 me3 indicates H3K27 me3.

Our results extend previous understanding by relating histone modifications to defined regions of pluripotency genes. Some changes, including increased phosphorylation and decreased acetylation, seem to be related to the general transcriptional activation characteristics of oocyte GVs (Figure 7). However, H3K4 methylation is likely to be specifically related to pluripotency gene activation. This is because this change is seen in the regulatory regions of these genes and is more pronounced in DmES nuclei that are efficiently reprogrammed than in 10T1/2 nuclei (Figure 7). It is possible that a lack of H3K27 me3 may also be important in reprogramming by oocytes.

We comment on an unusual histone change associated with nuclear transfer. We see that histone H2A.Z initially vanishes but reappears in 10T1/2 nuclei after nuclear transfer. H2A.Z extinction or replacement may reload H2A.Z onto chromatin, such that it can establish a specialized chromatin state necessary for the proper execution of developmental gene expression programs. The likely downstream genes of H2A.Z have been described [20]. These may cooperate with PcG proteins [20], or may be specially related to germ cell development [21]. There may also be some interplay with DNA methylation since H2A.Z can protect genes from DNA methylation [22].

The use of amphibian oocytes and eggs for analyzing transcriptional reprogramming has great potential value because of the huge amount of material available compared to oocytes and eggs of mammals [23]. The eventual use of extracts and their depletion may provide a productive route by which to identify reprogramming components [24,25]. The characterization of epigenetic changes associated with reprogramming in living oocytes, as described here, provides valuable information by which to guide the development of *in vitro *extracts.

## Conclusion

This work shows that mouse somatic cell nuclei, when transplanted to Xenopus oocytes, undergo multiple histone modifications. Among these modifications, H3K4 me3 is very likely to be important for the efficient reprogramming of mouse somatic nuclei by Amphibian oocytes. Aurora B kinase is required for H3S10 phosphorylation in transplanted somatic cell nuclei.

## Methods

### Cell culture and DmES cells in tissue culture

C3H/10T1/2 (10T1/2) cells were grown in Deulbecco's modified eagle medium containing 10% fetal bovine serum (FBS), 50 μg/ml of penicillin and streptomycin at 37°C and 5% CO_2_. Mouse embryonic stem (mES) cells were grown in gelatinized flasks in Glasgow minimum essential medium (GMEM) containing 20% FBS (qualified for ES cell culture), 50 ug/ml of penicillin and streptomycin, 1 mM sodium pyruvate, 0.1 mM non-essential amino acids, 0.1 mM 2-mercaptoethanol and 1000 U/ml leukaemia inhibitory factor. These mES cells are feeder free. mES cells were differentiated into neural cells in differentiation medium (GMEM containing 20% FBS, 50 μg/ml of penicillin and streptomycin, 1 mM sodium pyruvate, 0.1 mM non-essential amino acids, and 5 μM retinoic acid). mES cells usually differentiated into neural cells, neurons and glial cells, after 3 days in the differentiation medium. These differentiated mES cells were maintained in the differentiation medium until they were harvested for nuclear injection.

### Oocyte preparation and injection and cell permeabilization

Oocytes of *X. laevis *were prepared and treated as described before [12]. For these experiments we have aimed to inject 200 nuclei per oocyte. We find that 80% injected nuclei are usually deposited in the GV.

### Gene expression analysis on transplanted somatic cell nuclei by RT-PCR

RNA from four injected oocytes was isolated using an RNA isolation kit (Qiagen, CA, USA). cDNA was generated using Superscript III reverse transcriptase (Invitrogen, CA, USA). We used gene specific primers for synthesizing first strand DNA. We added 10 mM dithiothreitol, 1 mM dNTPs, 0.2 mg/ml bovine serum albumin and 40 units RNase inhibitors and incubated them at 42°C for 1 h. A part of the sample was diluted (1:10, vol/vol) by DNase/RNase free water and used immediately for polymerase chain reaction (PCR).

PCR was performed for 35 cycles under conditions of 95°C (30 s), 58°C (30 s) and 72°C (90 s) following a preheating step at 95°C for 15 min to amplify embryo-expressed genes but was performed for 25 cycles to analyse the GAPDH gene. Hot Start *Taq *polymerase was used in the presence of 25 mM MgSO_4_, 10 mM dNTPs and 10 nM gene specific primers in the buffer supplied by a company. Q-solution was added to the samples to amplify Oct4 or Sox2. PCR products were separated by electrophoresis on a 1.5% agarose gel. The primer sequences for this study are

Sox2 forward: GGAGTGGAAACTTTTGTCCGAGAC, Sox2 reverse: TGGAGTGGGAGGAAGAGGTAACC, Oct4 forward: GTGAGCCGTCTTTCCACCAG, Oct4 reverse: TTCTCCAACTTCACGGCATT, Sall4 forward: GGGGCTAAAATTTCCCAACT, Sall4 reverse: CTCCTCCCAGTTGATGTGCT, β-globin forward: GCTGGTTGTCTACCCTTGGA, β-globin reverse: ATCCACATGCAGCTTGTCAC, GAPDH forward: TCAACGACCCCTTCATTGAC, GAPDH reverse: ATGCAGGGATGATGTTCTGG.

### Immunofluorescent staining of injected nuclei

Injected oocytes were collected at 0, 12, 24, 48 or 72 h after nuclear transfer. The injected oocytes at zero hour were dissected within 1 min after the injection. Approximately 50 injected oocytes were harvested at each time point. Germinal vesicles from the injected oocytes were separated in isolation buffer (20 mM Tris/HCl pH7.5, 0.5 mM MgSO_4_, 140 mM KCl). The isolated GVs which contained injected somatic cell nuclei were transferred to 1 ml of 4% paraformaldehyde solution and fixed for 10 min. After the paraformaldehyde solution was removed, they were permeabilized in 1 ml of permeabilization buffer (0.5% Triton X-100 solution in phosphate buffered saline (PBS) for 5 min and washed three times with 1 ml of Washing/Blocking buffer (5% FBS, 0.2% Tween-20 in PBS). They were incubated in the same solution for at least 1 h for blocking. The injected somatic cell nuclei in GVs were resuspended in approximately 50 μl of washing/blocking buffer and stained by primary antibodies against the desired antigens (typically 1/100 or 1/200 dilution, depending on antibodies) over night. After staining, they were washed three times with washing/blocking buffer to remove excess primary antibodies and resuspended in 50 μl of washing/blocking buffer. They were then stained by secondary antibodies which were conjugated with alexa fluor (Molecular Probes, Oregon, USA; 1/200 dilution). They were washed three times with washing/blocking buffer and counterstained with DAPI (Invitrogen). Antibodies were provided by AB and TK, or purchased from Abcam.

### Western blotting of histones in nuclei from differentiated cells injected into germinal vesicles of X. oocytes or in nuclei treated with high speed X. oocyte extracts

Injected oocytes were collected and dissected in isolation buffer described above. The isolated GVs with injected cell nuclei were transferred to a tube with 1 ml of non-denaturing washing buffer (150 mM Tris-HCl ph7.5, 50 mM NaCl, 150 mM KCl). The GVs were vigorously vortexed for about 5 s in order to destroy GV membranes so that transplanted 10T 1/2 nuclei could be separated from the GVs. The nuclei were pelleted by a 16,000 g centrifugation for 3 min at room temperature. The nuclear pellets were washed twice with the non-denaturing washing buffer to remove any contaminants from GVs. The nuclear pellets recovered from GVs were lysed in high salt radioimmunopreciptation buffer (25 mM Tris-HCl pH7.6, 1% NP-40, 1% sodium deoxycholate, 0.1% sodium dodecyl sulphate (SDS), 2.5 M NaCl) with a protease inhibitor cocktail (Roche). The pellets were vigorously vortexed in the buffer at 4°C for 10 min and membranes were separated by a 13.2 krpm centrifugation at 4°C for 30 min.

Approximately 20,000 10T 1/2 cell nuclei were incubated in 20 μl of high speed oocyte extracts or in buffer for 0, 1 or 2 h at 18°C (the concentration of 10T1/2 cell nuclei in extracts was 1,000/μl). The nuclei were spun down with a 10 k rpm centrifugation at room temperature after the desired incubation. The nuclear pellets were washed with non-denaturing buffer three times with a 16,000 g centrifugation after each washing. These lysates were used for Western blotting.

### ChIP analysis on cell nuclei recovered from X. laevis germinal vesicles or injected oocytes

Approximately 200 10T1/2 cell nuclei or DmES cell nuclei were injected into *X. laevis *oocyte GVs and were collected at the desired times such as 0, 24, 48 or 72 h after nuclear transfer. Whole injected oocytes were lysed in a MNase digestion buffer (0.32 M Sucrose, 50 mM Tris-HCl pH 9.5, 4 mM MgCl_2_, 1 mM CaCl_2_, 0.1 mM phenylmethanesulphonylfuoride). The pH of Tris-HCl in this modified MNase digestion buffer is high because oocyte lysates are acidic and we used a limited quantity of the buffer so as not to dilute histones in samples (50 whole injected oocytes in 100 μl buffer). Therefore, Tris-HCl pH 9.5 was required to maintain oocyte lysates containing injected nuclei at around pH 7.5. The chromosomes in nuclear pellets were digested by micrococcal nuclease (MNase, 0.1 units/sample) at 18°C for 10 min in order to obtain mononucleosomes. The cell permeabilization step was omitted because these cells were already permeabilized before injection. 1 μl of 0.5 M EDTA and 1 μl of NP-40 was added to a 100 μl sample solution in order to stop micrococcal nuclease activities. The pellets were shaken gently for 30 min at 4°C to lyse nuclei completely. Samples were spun down and only supernatants were collected as a soluble chromatin fraction. Primary antibodies were added to the chromatin solution and incubated at 4°C over night. Non-immune rabbit IgG was used in the preliminary experiments. ChIP by histone H3 antibodies was used as a histone precipitation control instead of conducting sequential ChIPs. Protein A conjugated magnetic beads were added to the solution in order to precipitate the histone-antibody complexes. The histone-antibody complexes were washed extensively nine times with washing buffers containing a different concentration of salt (first washing buffer, three times with 75 mM NaCl; second washing buffer, three times with 125 mM NaCl; third washing buffer, three times with 175 mM NaCl in Tris-HCl pH 7.5 and 10 mM EDTA). The histone-antibody complexes were then digested by proteinase K overnight in elution buffer (50 mM Tis-HCl pH 7.5, 50 mM NaCl, 0.1 mM PMSF, 5 mM EDTA, 1% SDS). The free DNA was eluted in elution buffer. The eluted DNA (ChIP DNA) was purified by phenol chloroform extraction and ethanol precipitation method.

PCR was performed by real-time PCR (Applied Biosystems, CA, USA) for 40 cycles under conditions of 95°C (15 s), 58°C (60 s), and 60°C (60 s) followed by steps at 95°C for 15 s, 60°C for 60 s, 95°C for 15 s and 60°C for 15 s. The primer sequences for this study are from references [26] and [27].

Sox2 promoter region forward: CCATCCACCCTTATGTATCCAAG, Sox2 promoter region reverse: CGAAGGAAGTGGGTAAACAGCAC, Sox2 downstream regulatory region forward: CAGGTTCCCCTCTAATTAATGC, Sox2 downstream regulatory region reverse: CTGTGCTCATTACCACGTGAA, Sox2 gene coding region forward: GGAGCAACGGCAGCTA, Sox2 gene coding region reverse: GTAGCGGTGCATCGGT, Oct4 promoter region1 forward: GGCTCTCCAGAGGATGGCTGAG, Oct4 promoter region1 reverse: TCGGATGCCCCATCGCA, Oct4 gene coding region forward: CCTGCAGAAGGAGCTAGAACA, Oct4 gene coding region reverse: TGTGGAGAAGCAGCTCCTAAG, Sall4 promoter region forward: ATGCTGGGCCTTGTAGTCC, Sall4 promoter region reverse: ATCTGAGCCCGGATGCTAAT, β-globin promoter region forward: CTGCTCACACAGGATAGAGAGGG, β-globin promoter region reverse: GCAAATGTGAGGAGCAACTGTC, β-globin gene coding region forward: TCTACAGTTATGTTGATGGTTCTTCCA, β-globin gene coding region reverse: CAGGACAATCACGATCATATTGC.

The analysis of ChIP results was made as follows. The immediate results of real-time PCR were corrected by subtraction of the IgG background. These values were then adjusted so that the number 1.0 was allocated in each case to the T0 point, other values being changed in proportion. Finally the results were corrected by dividing each value by that obtained for the H3 histone control.

### Immunodepletion of Aurora B from oocyte extracts

Fifty microlitres of antibodies against Aurora B (Santa Cruz, CA, USA) or non-immune mouse IgG were added to 100 μl of oocyte extracts for 1 h at 4°C and protein A beads (Invitrogen) were added to precipitate antibodies for 30 min.

## Abbreviations

BSA: bovine serum albumin; ChIP: chromatin immunoprecipitation; DmES: differentiated mouse ES; ES: embryonic stem; FBS: fetal bovine serum; GMEM: Glasgow minimum essential medium; GV: germinal vesicle; m ES: mouse ES; PBS: phosphate buffered saline; PCR: polymerase chain reaction; PMSF: phenylmethanesulphonylfluoride; RIPA: radioimmunopreciptation assay; RT-PCR: reverse transcriptase polymerase chain reaction; SDS: sodium dodecyl sulphate.

## Competing interests

The authors declare that they have no competing interests.

## Authors' contributions

KM and JBG conceived of this study. KM designed and conducted all experiments, as well as analysing and interpreting the data. AJB and TK provided antibodies. KM and JBG wrote the paper. AJB reviewed the paper and gave critical and important intellectual comments.

## Supplementary Material

Additional file 1 **Kinetics of histone modification changes in somatic nuclei injected to germinal vesicles**. The intensity of immunofluorescent staining of modified histones is shown diagrammatically. Each histone modification is categorized as phosphorylation, acetylation, H2A.Z or methylation. Each modification is indicated on the left side of the graph and the trend of its change is shown on the right side (increase, decrease). Click here for file

Additional file 2 **The regulatory regions of Sox2, Oct4, Sall4, and β-globin **[26,27]**used for chromatin immunoprecipitation analysis**. The arrow indicates the transcription start site. Upstream sequences are shown as - nucleotides and downstream positions are indicated as + nucleotides or KG. Click here for file

Additional file 3 **Chromatin immunoprecipitation analyses of histone modifications in 10T1/2 cell nuclei transplanted to germinal vesicles**. Histone methylations, phosphorylations and acetylations in the gene coding regions of Sox2 (a, b and c), Oct4 (d, e and f) and β-globin (g, h and i) in transplanted 10T1/2 nuclei were analyzed. H3K4 me2, H3T3ph and H3K9ac are shown in blue; H3K4 me3, H3T6ph and H3K14ac are shown in red; H3K27 me3, H3T11ph and H4K16ac are shown in light green. The *Y *axis is the fold enrichment of modified histone over non-modified histone H3. The *X *axis is incubation time after nuclear transfer. Click here for file
