# Supplementary figures and images for: Histone H3 lysine 4 methylation is associated with the transcriptional reprogramming efficiency of somatic nuclei by oocytes
Source: Epigenetics Chromatin. 2010 Feb 4;3:4. doi: 10.1186/1756-8935-3-4 (PMC2829560; doi:10.1186/1756-8935-3-4)

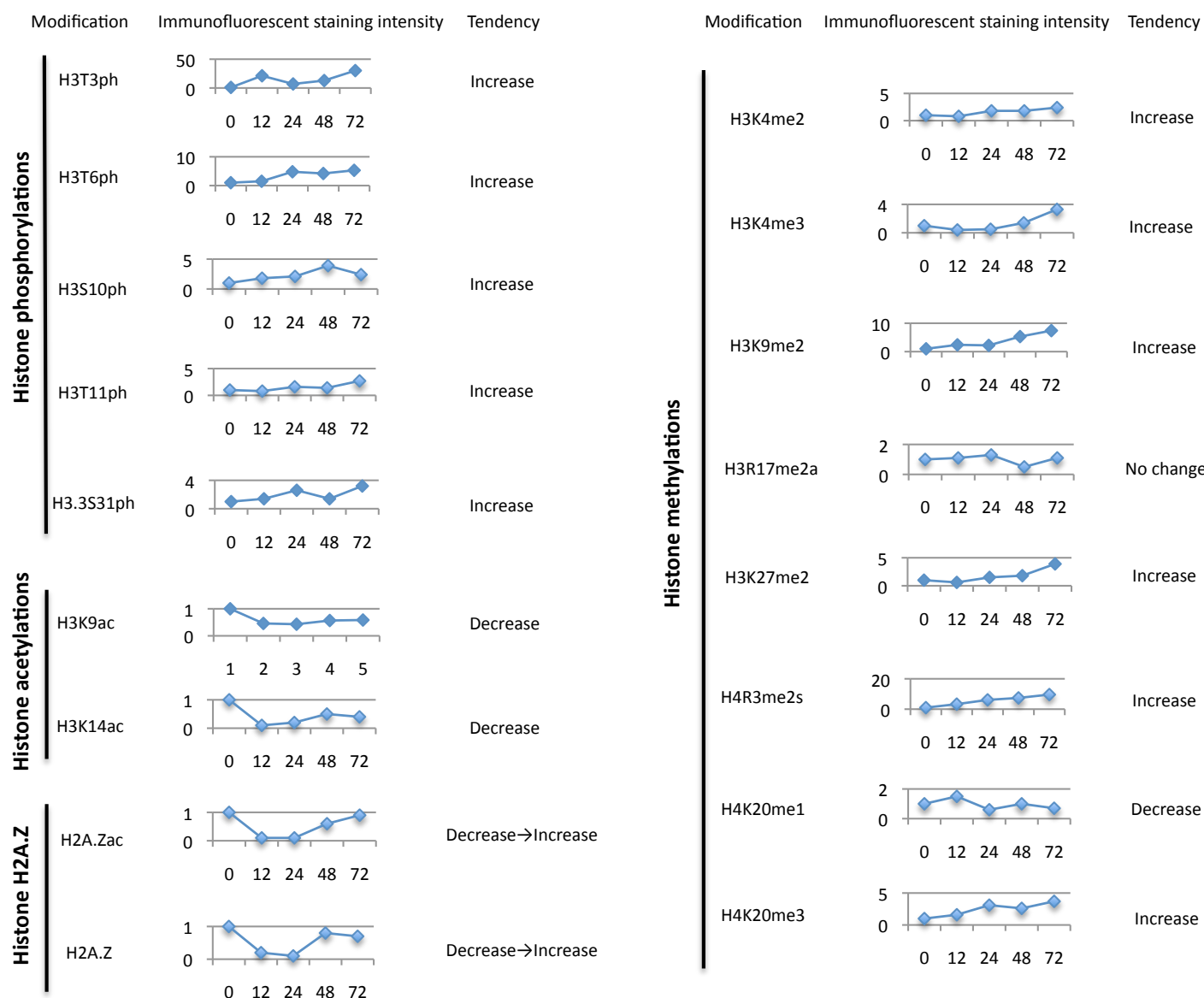

Additional file 1

Supplement: Additional file 1 — Kinetics of histone modification changes in somatic nuclei injected to germinal vesicles. The intensity of immunofluorescent staining of modified histones is shown diagrammatically. Each histone modification is categorized as phosphorylation, acetylation, H2A.Z or methylation. Each modification is indicated on the left side of the graph and the trend of its change is shown on the right side (increase, decrease). [file 1756-8935-3-4-S1.PDF]

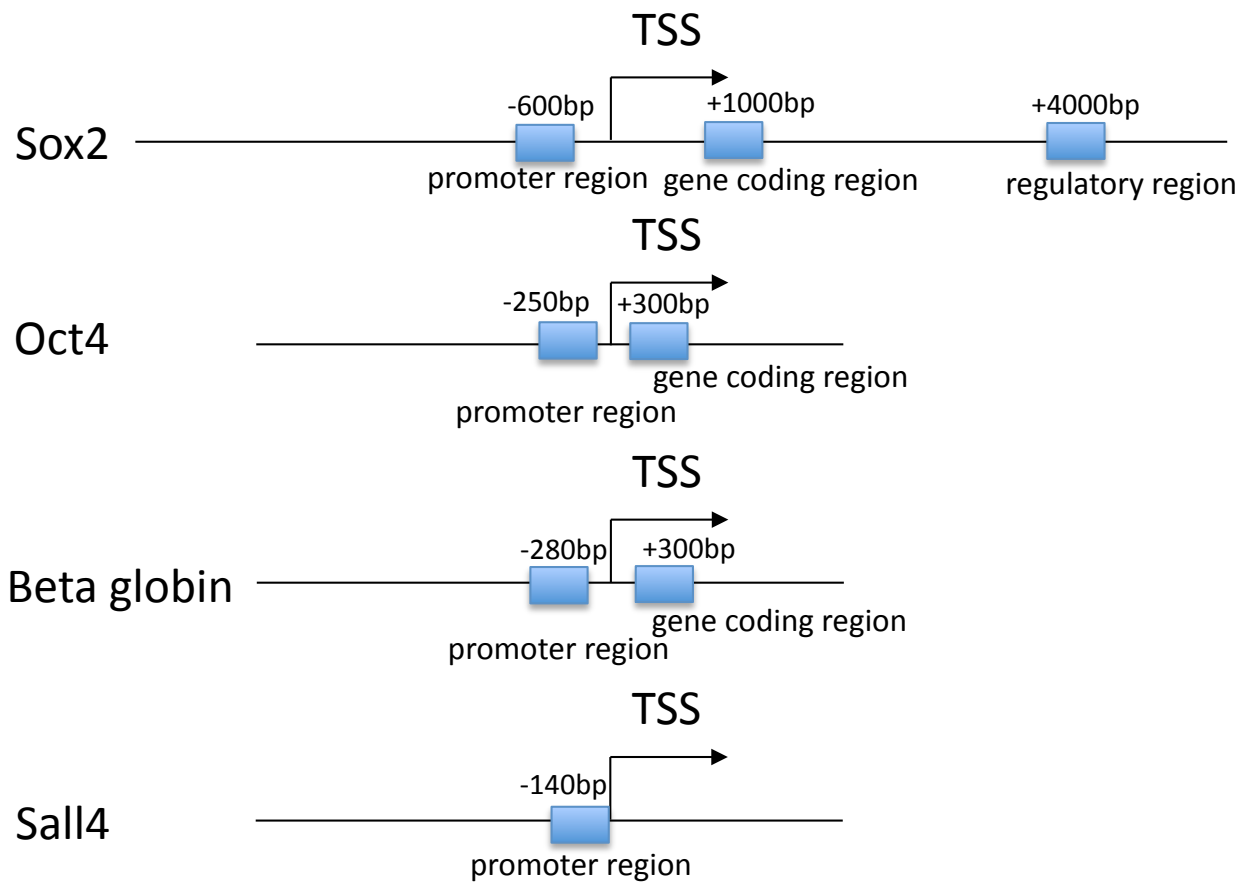

Additional file 2

Supplement: Additional file 2 — The regulatory regions of Sox2, Oct4, Sall4, and β-globin [26,27]used for chromatin immunoprecipitation analysis. The arrow indicates the transcription start site. Upstream sequences are shown as - nucleotides and downstream positions are indicated as + nucleotides or KG. [file 1756-8935-3-4-S2.PDF]

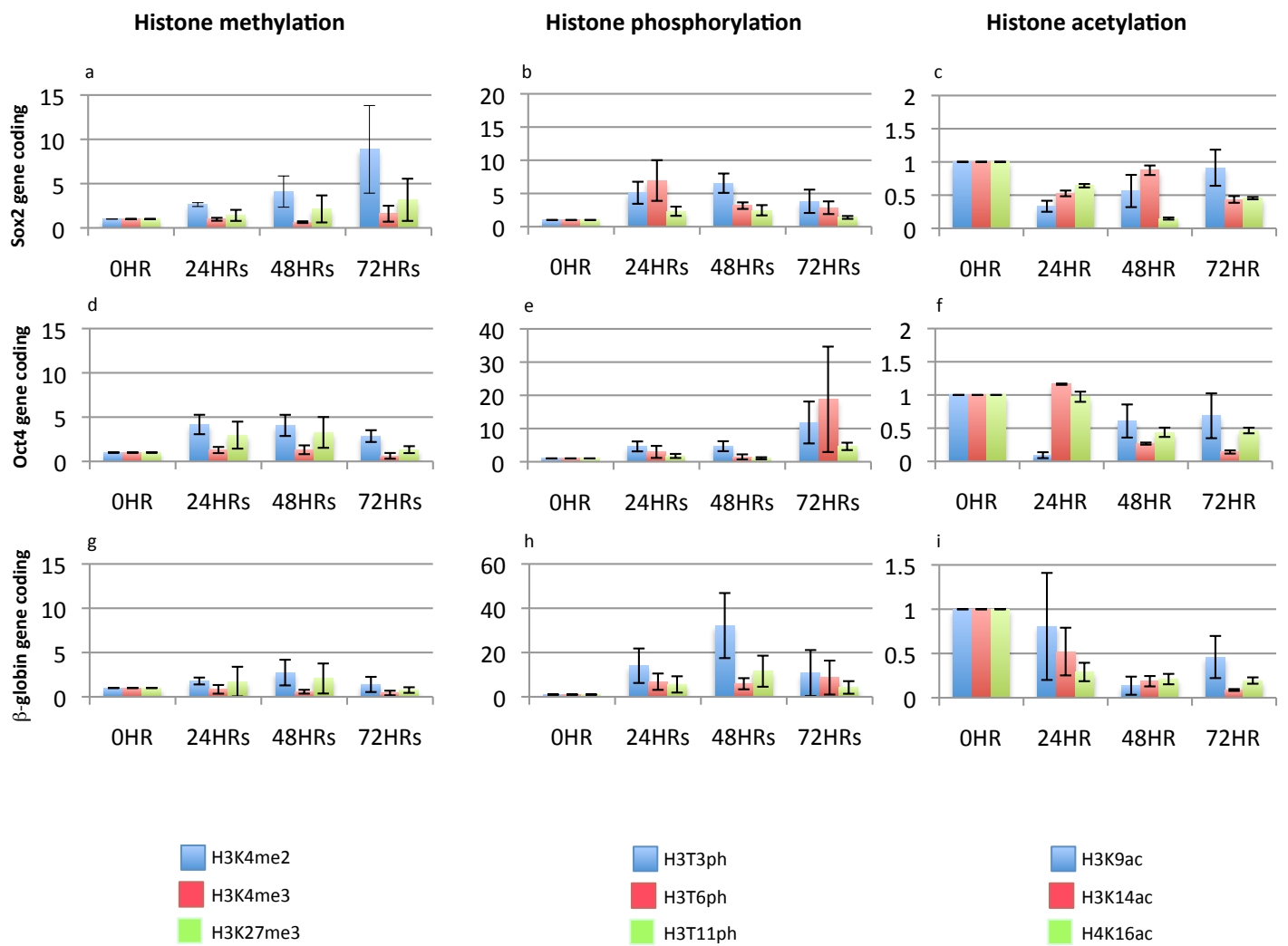

Additional file 3

Supplement: Additional file 3 — Chromatin immunoprecipitation analyses of histone modifications in 10T1/2 cell nuclei transplanted to germinal vesicles. Histone methylations, phosphorylations and acetylations in the gene coding regions of Sox2 (a, b and c), Oct4 (d, e and f) and β-globin (g, h and i) in transplanted 10T1/2 nuclei were analyzed. H3K4 me2, H3T3ph and H3K9ac are shown in blue; H3K4 me3, H3T6ph and H3K14ac are shown in red; H3K27 me3, H3T11ph and H4K16ac are shown in light green. The Y axis is the fold enrichment of modified histone over non-modified histone H3. The X axis is incubation time after nuclear transfer. [file 1756-8935-3-4-S3.PDF]
